# Integrated Role of Musculoskeletal Ultrasound in Piriformis Syndrome: A Case Series

**DOI:** 10.7759/cureus.109123

**Published:** 2026-05-18

**Authors:** Prithvi Sudhakar, Venkata Sai, Madhumita Sugumaran, Bathrenathh Balasubramanian, Niranjana Sugumaran

**Affiliations:** 1 Department of Radiology, Sri Ramachandra Institute of Higher Education and Research, Chennai, IND

**Keywords:** botulinum toxin, deep gluteal syndrome, musculoskeletal ultrasound, piriformis syndrome, sciatic nerve, ultrasound-guided injection

## Abstract

Piriformis syndrome (PS) is an uncommon entrapment neuropathy in which the sciatic nerve is compressed by the piriformis muscle, producing gluteal and radiating leg pain that closely mimics lumbar disc disease and other causes of sciatica, making accurate diagnosis a persistent clinical challenge. Musculoskeletal (MSK) ultrasound has emerged as a valuable imaging modality in this setting, offering real-time, dynamic assessment of muscle morphology, sciatic nerve architecture, and regional vascularity without the use of ionizing radiation. In this retrospective case series of three patients diagnosed with PS at a tertiary care centre in India over a two-year period, high-resolution MSK ultrasound with dynamic provocative maneuvers was employed to evaluate sciatic nerve behaviour in relation to the piriformis muscle. Dynamic imaging successfully demonstrated nerve compression in all three cases, with measurable changes in nerve caliber at the site of entrapment compared to adjacent unaffected segments, providing objective morphological evidence to support the clinical diagnosis. Following diagnostic confirmation, ultrasound-guided injection therapy was administered with complete positional accuracy; two patients received botulinum toxin A to achieve muscle relaxation and sustained nerve decompression, while one patient received a corticosteroid and local anesthetic combination to address inflammatory pain. All three patients achieved complete symptomatic resolution at four-week follow-up, underscoring the therapeutic efficacy of image-guided intervention when the diagnosis is precise. These cases collectively illustrate that MSK ultrasound, through its capacity for real-time dynamic evaluation and procedural guidance, represents a versatile and indispensable tool in the diagnosis and minimally invasive management of PS within a multidisciplinary clinical framework.

## Introduction

Piriformis syndrome (PS) is a peripheral neuromuscular disorder in which the piriformis muscle irritates or compresses the sciatic nerve, producing pain, paresthesia, and functional limitation in the gluteal region and lower limb. Although historically considered a rare condition, increasing recognition of deep gluteal syndrome (DGS) and improved imaging techniques have led to greater diagnostic awareness [1,2]. The true prevalence of PS remains uncertain, partly because it mimics lumbar disc herniation, sacroiliac joint dysfunction, and other causes of sciatica, making diagnosis challenging on clinical grounds alone [1].

The piriformis muscle originates from the anterior sacral surface and inserts onto the superior aspect of the greater trochanter of the femur. It functions primarily as an external rotator of the hip in extension; however, when the hip is flexed beyond approximately 60 degrees, its mechanical axis shifts such that it acts as an internal rotator and abductor - a biomechanical duality clinically relevant to understanding how certain postures and movements provoke nerve compression in PS.

Its anatomical relationship with the sciatic nerve is variable: in the most common configuration (Beaton and Anson Type I), the undivided sciatic nerve exits the greater sciatic foramen inferior to the muscle. Aberrant relationships - including passage of nerve divisions through or above the muscle - occur in up to 20% of individuals and predispose to entrapment [2,3].

Conventional diagnostic imaging, including plain radiography and magnetic resonance imaging (MRI), offers limited dynamic information. Musculoskeletal (MSK) ultrasound, by contrast, allows real-time assessment of muscle contraction, nerve displacement, and perineural vascularity. The ability to elicit and observe nerve compression during provocative hip rotation maneuvers provides diagnostic information unavailable through static modalities [1,3]. Furthermore, ultrasound guidance enables precise delivery of therapeutic agents - corticosteroids, local anesthetics, or botulinum toxin - directly into the piriformis muscle or the perineural space adjacent to the sciatic nerve.

This case series presents three patients with confirmed PS, illustrating the integrated diagnostic and therapeutic role of MSK ultrasound at a tertiary care institution in India.

## Case presentation

**Figure 1:**
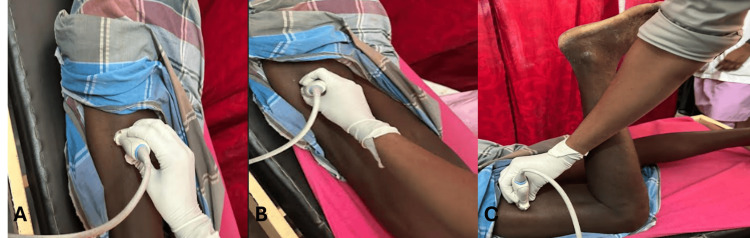
Technique for assessment of piriformis syndrome (A) Patient position: The patient is lying prone with the hips and knees extended straight. (B) Place the transducer perpendicular to the femur shaft at the greater trochanter, slide medially, and rotate to align with the piriformis muscle fibres for an oblique/long axis view. (C) Flex knee to 90 degrees, rotate hip internally/externally for dynamic assessment of gluteus maximus and piriformis muscles.

This is a retrospective case series conducted at a tertiary care hospital in India over a two-year study period (2023 to 2025). Institutional ethical approval was obtained, and informed consent was documented for each patient prior to the ultrasound examination and injection procedure. All three patients were examined in the prone position using a high-frequency linear array transducer (GE LOGIQ (GE HealthCare, Chicago, Illinois, USA) and Samsung V8 systems (Samsung Medison Co., Ltd., Seoul, South Korea)). The piriformis muscle, sciatic nerve, ischial tuberosity, and greater sciatic notch were systematically identified. Dynamic provocative testing was performed with the knee flexed to 90° during hip internal and external rotation (Figure 1). Ultrasound-guided injections were performed in-plane under real-time visualization, with clinical response assessed at four-week follow-up.

Case 1

Presenting Complaint

A patient presented with right gluteal region pain of six months' duration.

Ultrasound Findings

The right ischial tuberosity and piriformis muscle appeared normal in echotexture and caliber. The right sciatic nerve was measured at 0.32 cm at the site of compression beneath the piriformis muscle, increasing to 0.39 cm just beyond the point of compression, and measuring 0.41 cm at the mid-thigh level. Dynamic internal and external hip rotation elicited pain with demonstrable sciatic nerve compression by the piriformis muscle, consistent with PS.

Intervention

**Figure 2:**
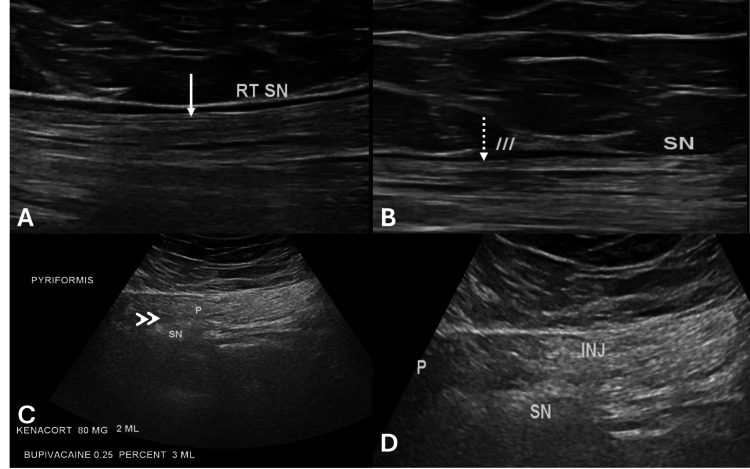
Ultrasound-guided evaluation and injection therapy in piriformis syndrome - Case 1 (A) Normal diameter of the sciatic nerve proximal to the site of compression (solid white arrow). (B) Compression of the sciatic nerve below the piriformis muscle (dotted white arrow). (C) Ultrasound-guided injection of 80 mg (2 mL) triamcinolone (Kenacort) with 3 mL of 0.25% bupivacaine into the piriformis muscle (double arrowhead). (D) Post-injection image. SN: sciatic nerve; P: piriformis muscle

Ultrasound-guided injection of triamcinolone acetonide 80 mg (2 mL) combined with 0.25% bupivacaine (3 mL) was administered into the piriformis muscle. Injection accuracy was 100% (Figure 2).

Outcome

Complete resolution of symptoms was observed at the four-week follow-up.

Case 2

Presenting Complaint

A patient presented with radiating pain in the left gluteal region of four months' duration.

Ultrasound Findings

The right sciatic nerve measured 0.19 cm with normal echogenicity. The left sciatic nerve at the site of compression below the piriformis muscle demonstrated altered echogenicity with a focal constriction, measuring a maximum of 0.13 cm at the compression point and 0.17 cm proximal to it; the mid-thigh caliber measured 0.22 cm. Probe tenderness was elicited in the left gluteal region. Dynamic assessment confirmed left sciatic nerve compression by the piriformis muscle with reproduction of the patient's pain.

Intervention

**Figure 3:**
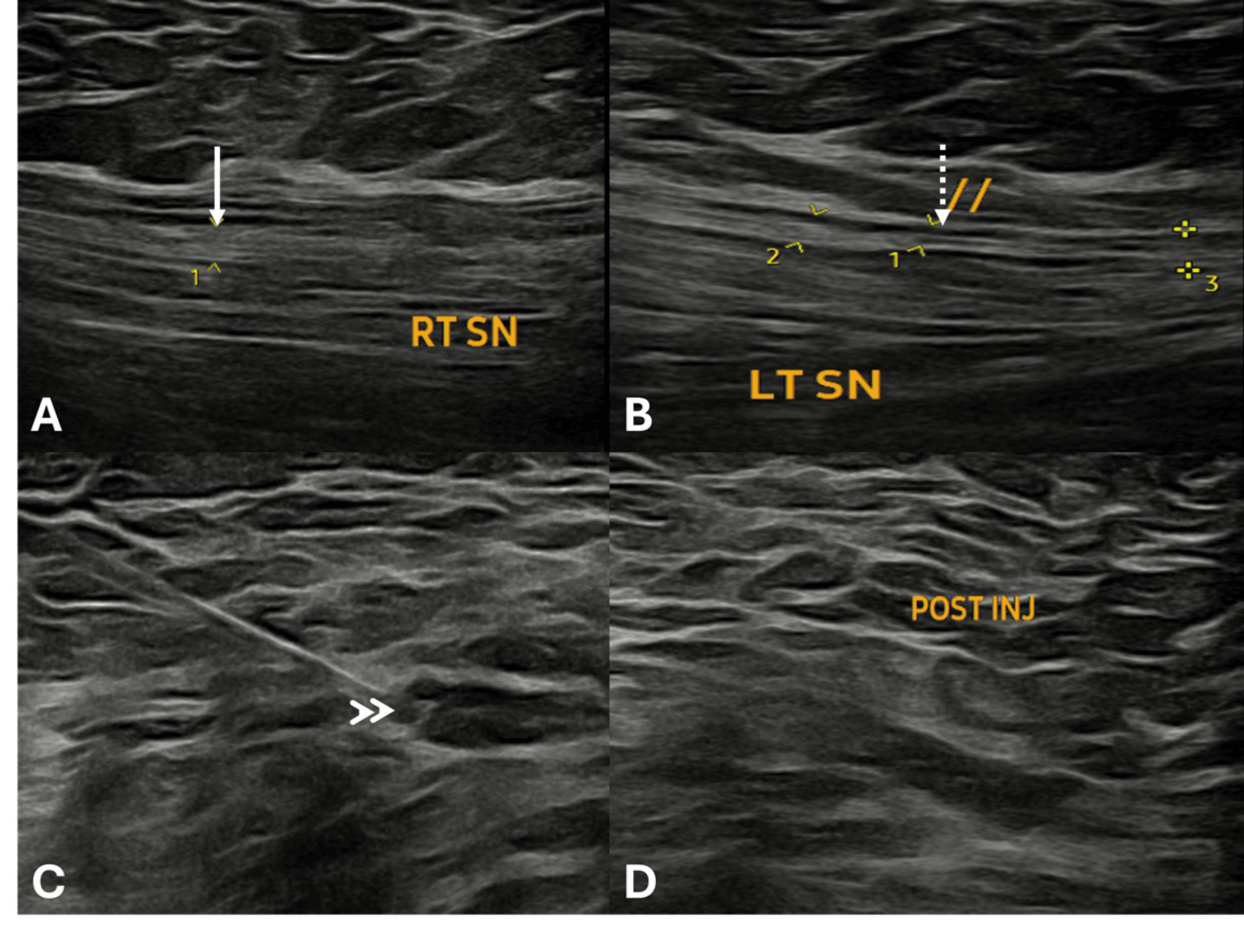
Ultrasound-guided botulinum toxin injection for piriformis syndrome - Case 2 (A) Normal right sciatic nerve (solid white arrow). (B) Compression of the left sciatic nerve below the piriformis muscle with altered echogenicity (dotted white arrow). (C) Ultrasound-guided injection of botulinum toxin type A (100 units) mixed with saline into the left gluteal region adjacent to the left sciatic nerve near the piriformis muscle (double arrowhead). (D) Post-injection image. SN: sciatic nerve

Ultrasound-guided injection of botulinum toxin type A (BTX-A) (100 units in normal saline) was administered adjacent to the left sciatic nerve near the piriformis muscle. Injection accuracy was 100% (Figure 3).

Outcome

Complete resolution of symptoms was observed at the four-week follow-up.

Case 3

Presenting Complaint

A patient presented with pain and numbness in the right gluteal region and leg of five months' duration.

Ultrasound Findings

In the right gluteal region, the sciatic nerve below the greater sciatic notch and the piriformis muscle measured a maximum of 0.28 cm and appeared compressed. The right piriformis muscle was edematous with mildly altered echotexture. For comparison, the contralateral left sciatic nerve at the same level measured 0.37 cm with normal morphology. Dynamic assessment reproduced pain with sciatic nerve compression by the right piriformis muscle.

Intervention

**Figure 4:**
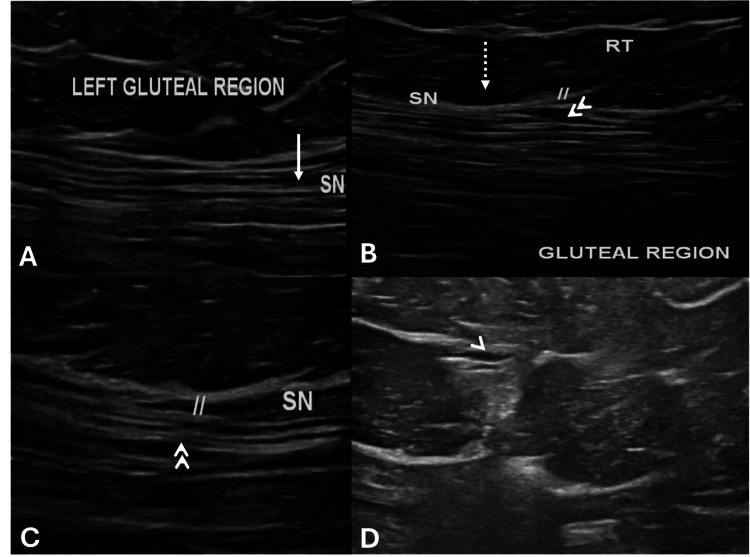
Ultrasound findings and botulinum toxin injection in piriformis syndrome - Case 3 (A) Normal left sciatic nerve (solid white arrow). (B and C) Right gluteal region showing an edematous piriformis muscle with mildly altered echotexture (dotted white arrow) and compression of the sciatic nerve below the piriformis muscle (double arrowhead). (D) Ultrasound-guided injection of botulinum toxin type A (100 units) mixed with saline administered slowly near the compressed sciatic nerve region (arrowhead). SN: sciatic nerve

Ultrasound-guided injection of BTX-A (100 units in normal saline) was administered slowly adjacent to the compressed right sciatic nerve. Injection accuracy was 100% (Figure 4).

Outcome

Complete resolution of symptoms was observed at the four-week follow-up.

A summary of the clinical presentation, ultrasound findings, intervention, and outcomes of all three cases is provided in Table 1.

**Table 1:** Summary of case series findings and outcomes

Parameter	Case 1	Case 2	Case 3
Chief complaint	Right gluteal pain × six months	Left gluteal radiating pain × four months	Right gluteal pain and numbness × five months
Side affected	Right	Left	Right
Dynamic assessment	Compression of the right sciatic nerve by the piriformis; pain elicited	Compression of the left sciatic nerve by the piriformis; pain elicited	Compression of the right sciatic nerve by the piriformis; pain elicited
Sciatic nerve diameter at compression	0.32 cm	0.13 cm (constriction)	0.28 cm
Sciatic nerve diameter distal to compression	0.39 cm	0.22 cm (mid-thigh)	0.37 cm (contralateral reference)
Piriformis muscle findings	Normal echotexture	Normal (right); altered echogenicity with nerve constriction (left)	Edematous with mildly altered echotexture
Drug used	Triamcinolone 80 mg (2 mL) + bupivacaine 0.25% (3 mL)	Botulinum toxin type A 100 units in saline	Botulinum toxin type A 100 units in saline
Injection accuracy	100%	100%	100%
Response at four-week follow-up	Complete resolution of symptoms	Complete resolution of symptoms	Complete resolution of symptoms

## Discussion

This case series demonstrates the central role of MSK ultrasound in both diagnosing and managing PS, a condition whose clinical recognition has historically been hampered by its significant overlap with lumbar radiculopathy, sacroiliac joint dysfunction, and other causes of gluteal and sciatic pain [1,2]. Increasing awareness of DGS - a broader anatomical construct encompassing all causes of extra-spinal sciatic nerve compression within the gluteal space - has repositioned PS as one component of a wider diagnostic spectrum rather than an isolated entity [3,4]. Hernando et al. and Martin et al. have been instrumental in formalizing DGS as a clinically distinct syndrome, emphasizing that piriformis-related compression must be differentiated from other causes of subgluteal nerve entrapment, including fibrous bands, gemelli-obturator complex pathology, and ischiofemoral impingement [3,4]. Our three cases, all presenting with posterior gluteal and leg pain without imaging evidence of lumbar disc pathology, are consistent with this revised conceptual framework.

The diagnosis of PS remains contentious in the absence of a universally accepted gold-standard test. Clinical criteria proposed by Fishman et al. - incorporating provocation with the flexion, adduction, internal rotation (FLAIR) maneuver and electrodiagnostic confirmation of H-reflex delay - provide a structured diagnostic framework with reported sensitivity of 0.881 and specificity of 0.832 [5]. However, the systematic review by Hopayian et al. identified substantial heterogeneity in the diagnostic criteria applied across published studies, with the FAIR test representing the most consistently reported clinical sign despite limited standardization [6]. Imaging-based diagnosis has evolved in parallel: while plain radiography has no diagnostic role, MRI can demonstrate piriformis muscle hypertrophy, T2 signal alteration, and perineural changes along the sciatic nerve - findings described by Petchprapa et al. in a radiographic review of gluteal nerve entrapment syndromes [7]. The present series employed high-resolution MSK ultrasound with dynamic provocative maneuvers as the primary diagnostic modality, an approach validated by Vassalou et al. in a cross-sectional imaging study of 116 patients that demonstrated sonographic findings closely paralleling MRI in the identification of sciatic nerve compression at the piriformis level [8]. Critically, the dynamic capability of ultrasound - enabling real-time observation of nerve displacement, piriformis contraction, and pain reproduction during hip rotation - provides functional diagnostic information that neither MRI nor electrodiagnostics can replicate in isolation [1,9].

A key sonographic observation across all three cases was the demonstrable change in sciatic nerve caliber at and beyond the site of piriformis compression - a pattern analogous to the pseudoneuroma or proximal nerve swelling recognized in other peripheral entrapment neuropathies such as carpal tunnel syndrome and cubital tunnel syndrome [10]. In Case 1, the nerve measured 0.32 cm at the compression site and 0.41 cm at mid-thigh. In Case 2, a focal constriction to 0.13 cm was accompanied by altered echogenicity, suggesting significant perineural fibrosis consistent with chronic compressive neuropathy. In Case 3, comparison with the contralateral nerve (0.37 cm) against the affected side (0.28 cm) provided an internal caliber reference. These measurements align with the normative sciatic nerve diameter data reported by Cartwright et al. (mean 0.49 cm cross-sectional area at the mid-thigh) and Tagliafico et al., both of whom highlight that diameter and cross-sectional area measurements differ in reproducibility, with cross-sectional area using the ellipse formula demonstrating superior inter-rater reliability [11,12]. Future studies in this domain should standardize sciatic nerve measurements by cross-sectional area rather than linear diameter to enable meaningful inter-study comparison and facilitate normative reference ranges across age and body habitus strata.

The anatomical basis for entrapment in all three cases conformed to the Beaton and Anson Type I variant, wherein the undivided sciatic nerve passes inferior to the piriformis muscle - the configuration accounting for 80-90% of individuals [2]. Aberrant anatomical relationships, including passage of nerve divisions through or above the muscle, occur in up to 20% of the population and have been associated with increased susceptibility to entrapment, though clinical severity appears more closely correlated with the degree of dynamic compression during muscle activation than with the specific anatomical variant [2,8]. The biomechanical duality of the piriformis muscle - functioning as an external rotator in hip extension and transitioning to an internal rotator and abductor beyond approximately 60° of hip flexion - explains why certain postures and activities selectively provoke symptoms, a mechanism highlighted by Boyajian-O’Neill et al. in their clinical review of PS pathophysiology [13]. This functional anatomy underpins the rationale for provocative dynamic ultrasound assessment, which mimics the biomechanical stresses responsible for symptom generation.

Regarding the therapeutic approach, we employed two interventional strategies: a corticosteroid-local anaesthetic combination in Case 1 and BTX-A in Cases 2 and 3. Corticosteroid injections reduce perineural inflammation and have an established role in the management of nerve entrapment syndromes, with Benson and Schutzer reporting symptomatic relief in a cohort of patients with clinically confirmed PS following fluoroscopically guided injection [14]. The anti-inflammatory mechanism of corticosteroids is particularly relevant where an inflammatory component - evidenced by piriformis muscle edema and altered echotexture, as seen in Case 3 - contributes to nerve irritation. BTX-A operates through a distinct and complementary mechanism: temporary chemodenervation of the piriformis muscle reduces compressive tone on the sciatic nerve independent of any inflammatory pathway [15]. The randomized controlled trial by Fishman et al. - the most methodologically rigorous study to date in this field - demonstrated statistically significant superiority of BTX-A over corticosteroid-lidocaine injection in a double-blind crossover trial of 36 patients, with effect sizes favoring BTX-A for both pain reduction and functional improvement at 16 weeks [15]. Porta similarly reported favorable outcomes with BTX-A compared to methylprednisolone in a prospective comparative study [16]. Michel et al. reviewed the broader application of BTX-A in MSK pain and highlighted its utility in conditions where mechanical compression is the dominant pathophysiological mechanism, as is the case in PS [17]. The selection of corticosteroid versus BTX-A should therefore be individualized: where an inflammatory etiology predominates, corticosteroids offer a well-established, cost-effective intervention; where mechanical compression is primary - particularly in cases with prominent piriformis muscle dysfunction or previous corticosteroid failure - BTX-A is the preferred agent [13,15].

The precision of image-guided injection is a critical determinant of therapeutic outcome in PS, given the depth and anatomical complexity of the target region. Landmark-based injection into the piriformis muscle carries a substantial risk of misdirected drug delivery, with cadaveric studies suggesting accuracy rates as low as 30% using surface anatomy alone [18]. Fluoroscopic guidance improves accuracy but confers radiation exposure and cannot visualize soft tissues or the sciatic nerve directly. Computed tomography guidance offers excellent spatial resolution but is associated with a significant radiation dose and is poorly suited to dynamic assessment [18]. Ultrasound-guided injection has demonstrated accuracy rates comparable to CT guidance, as established in the systematic review by Misirlioglu et al., while eliminating radiation exposure and enabling real-time visualization of needle tip position, injectate spread, and the adjacent neurovascular structures [19]. Finnoff et al. similarly validated ultrasound-guided piriformis injection against fluoroscopic control, confirming equivalent anatomical accuracy [20]. The 100% injection accuracy achieved in all three cases in this series is consistent with these benchmarks and reflects the reproducibility of real-time ultrasound guidance in experienced hands.

The uniformly excellent outcomes observed in this series - complete symptom resolution in all three patients at four-week follow-up - must be interpreted in the context of the broader interventional literature, which reports more heterogeneous results. The systematic review by Hopayian et al. identified significant variability in treatment response across published series, attributable to inconsistent patient selection, heterogeneous diagnostic criteria, variable injection technique, and short follow-up durations [6]. The systematic review by Probst et al. similarly noted that while interventional treatments for PS generally yield short-term symptomatic benefit, evidence for durable long-term remission beyond 12 months is limited [21]. The favorable outcomes in our series likely reflect the stringent sonographic patient selection - all three cases satisfied both clinical and dynamic imaging criteria - as well as the precision of ultrasound-guided drug delivery. The small sample size and four-week follow-up window preclude conclusions about long-term efficacy or recurrence risk, and prospective studies with extended follow-up, validated functional outcome measures, and larger cohorts are needed to establish the comparative effectiveness of corticosteroid versus BTX-A injection under ultrasound guidance.

This series carries the inherent limitations of retrospective case series design: a small sample of three patients, the absence of a control group, no blinding, and a limited follow-up of four weeks. MRI was not performed in any case, precluding direct comparison with what many regard as the reference standard for soft tissue characterization and alternative diagnosis exclusion. Electrodiagnostic studies were similarly unavailable, limiting our ability to objectively quantify the degree of axonal injury or monitor neurophysiological recovery. Nerve caliber measurements were obtained as linear diameters rather than cross-sectional area, which may reduce reproducibility. Notwithstanding these limitations, the findings contribute incrementally to the growing evidence base supporting the integration of MSK ultrasound into the diagnostic and therapeutic algorithm for PS, particularly in resource-limited settings where MRI availability, cost, or waiting times represent a practical constraint [1,9].

## Conclusions

MSK ultrasound is a versatile, accessible, and radiation-free modality that enables objective dynamic diagnosis and real-time therapeutic guidance in PS. Dynamic assessment with provocative hip rotation maneuvers, combined with quantitative sciatic nerve caliber measurements at and beyond the compression site, provides reproducible diagnostic criteria that augment clinical assessment. Ultrasound-guided injection - whether using corticosteroids or BTX-A - achieves consistent and accurate drug delivery, resulting in excellent short-term clinical outcomes. These cases support the routine integration of MSK ultrasound into the diagnostic and therapeutic algorithm for PS.
